# Forensic shotgun pellet examination – material detection with dual-energy computed tomography

**DOI:** 10.1007/s00414-025-03553-8

**Published:** 2025-07-08

**Authors:** Mikael Brix, Juho-Antti Junno, Eveliina Lammentausta, Alina Junno, Timo Liimatainen, Jaakko Niinimäki, Juha Kiljunen, Petteri Oura

**Affiliations:** 1 Department of Diagnostic Radiology, Oulu University Hospital, Kajaanintie 50, Oulu, FI-90220 Finland; 2 Research Unit of Health Sciences and Technology, University of Oulu, Oulu, FI- 90014 Finland; 3 Department of Archaeology, University of Oulu, P.O. Box 8000, Oulu, FI-90014 Finland; 4 Cancer and Translational Medicine Research Unit, Medical Research Center Oulu, Oulu University Hospital and University of Oulu, P.O. Box 8000, Oulu, FI-90014 Finland; 5 Research Unit of Health Sciences and Technology, Medical Research Center Oulu, Oulu University Hospital and University of Oulu, P.O. Box 8000, Oulu, FI-90014 Finland; 6 Forensic Laboratory, Firearms Investigations, National Bureau of Investigation, P.O. Box 285, Vantaa, FI-01301 Finland; 7 Department of Forensic Medicine, University of Helsinki, P.O. Box 21, Helsinki, FI- 00014 Finland; 8 Forensic Medicine Unit, Finnish Institute for Health and Welfare, P.O. Box 30, Helsinki, FI-00271 Finland

## Abstract

Forensic shotgun wounds are relatively common worldwide. Shotguns are used for legal purposes such as hunting as well as for criminal activities. In addition, shotguns are used by military and law enforcement. Even though shotguns are often lethal from close distances, shotgun wounds require medical attention regardless of the shooting distance. As a result, patients with shotgun wounds are often treated in hospitals, and in lethal incidents, they are examined by forensic pathologists. Radiological imaging, computed tomography (CT) in particular, may constitute a part of the shotgun wound examination in both scenarios. CT may help to identify and locate the pellets and aid in wound characterization. It would be important to detect the pellet material as lead pellets, for example, are heavily toxic, and others, such as steel pellets may, as ferromagnetic, prevent some further imaging modalities such as magnetic resonance imaging from being employed. In this study, we wanted to experiment whether shotgun pellet material could be detected with radiological imaging utilizing dual-energy CT (DECT). Traditionally pellets are manufactured from heavily toxic lead, but due to environmental factors, other pellet materials such as steel, copper, tungsten, and bismuth are getting more and more popular. To conduct this study, various pellet materials were shot into ballistic gelatine blocks. The blocks then underwent DECT. Subsequently, each pellet was automatically segmented, and for these extracted pellets a dual-energy index (DEI) was computed. DEI values of the pellets were compared to determine whether this measure could be used to differentiate between the pellet materials. The DEI values (mean ± standard deviation) were 0.212 ± 0.006, 0.008 **±** 0.001, 0.187 **±** 0.002, 0.012 **±** 0.004, and 0.008 **±** 0.002 for steel, lead, copper, tungsten, and bismuth, respectively. The Wilcoxon rank sum test indicated that steel and copper pellets could be reliably differentiated from the other pellet materials. Our study demonstrated that DECT can reliably differentiate steel and copper shotgun pellets from other materials such as lead, tungsten, and bismuth. This capability may provide important additional information in medico-legal investigations and aid trauma centres treating patients with shotgun wounds.

## Introduction

Even just in the US, tens of thousands of people experience fatal and non-fatal gunshot injuries every year (Gani et al. 2017). Most of the non-fatal injuries leave the projectile or parts/fragments of the projectile retained in the body (Mushtaque et al. 2012; Kara et al. 2008). As projectile materials such as lead may be toxic, these fragments may be a high risk for the victims’ health and lead to long-term health problems (Nee et al. 2021).

Shotguns are popular, multi-purpose firearms. Shotguns are effective only from the close range. Commonly, the effective, deadly range of a shotgun is less than 50 m (Rowe and Hanson 1985). However, individual pellets may be dangerous even from a range of up to several hundred meters. Pellets may penetrate the skin and cause soft tissue injuries. Even minor injuries may constitute a health risk as retained pellets may elevate lead levels in the body (Weiss et al. 2017). Pellets may also migrate to other tissues, causing severe internal damage (Utkan et al. 2020).

Shotgun pellets are traditionally made from lead. However, as a toxic substance, lead shot has been banned from several uses especially in the hunting scene. Lead poisoning was a common cause of death in the past for waterfowl and their predators such as birds of prey (Golden et al. 2016). Thus, in the US, lead was first banned in waterfowling and in some states in all hunting. In waterfowling, lead is mainly replaced by steelshot, and nowadays, shotgun wounds requiring medical attention involve steel pellets more and more frequently (Byrne 2023). Being ferromagnetic, retained steel pellets may impose challenges for medical imaging in case certain modalities such as magnetic resonance imaging (MRI) are required.

Radiological examination of gunshot injuries is a standard procedure. An X-ray can reveal the radiopaque projectiles and their fragments (Pinotti et al. 2017). In the clinical context, gunshot victims are often scanned with clinical CT equipment to examine gunshot wounds and potential projectiles lodged in the body (Cañas et al. 2007). However, CT cannot directly detect projectile materials as the Hounsfield Units (HU) of different metals and even tissue types tend to overlap considerably. DECT and multi-energy CT, which allow the collection of more than two energy channels simultaneously, have been demonstrated to enable tissue quantification (Juntunen et al. 2020; Symons et al. 2017) and potentially, this methodology could be further utilized to distinguish other substances and materials from each other.

Postmortem dual-energy computed tomography (DECT) could aid in several potential scenarios involving shotgun pellets. For example, in homicide cases if the pellets cannot be physically retrieved for analysis by means of an immediate full medico-legal autopsy; in mass disasters if resources for full medico-legal autopsies are limited; and in routine autopsies as an auxiliary examination to quickly obtain a high coverage of pellets for digital analysis, especially in cases with several shotgun shell types present at the scene. In trauma centers, when treating gunshot injury victims and other patients with remnants of ballistic material embedded in their body, MRI may be problematic due to the risk of potential ferromagnetism if the retained ballistic material is unknown (Ditkofsky et al. 2020). It would thus be beneficial to be able to identify the material of the pellet to make conclusions about the potential ferromagnetism as well as toxicity.

Previous studies have already demonstrated the additional value of DECT against conventional CT in assessing the ferromagnetic properties of ballistic projectiles. In a study performed by Winklhofer and colleagues (2014), 25 bullets and two pellets were examined with DECT inside an anthropometric chest phantom. Gascho and colleagues (2020) on the other hand demonstrated the potential of extended CT scale to study projectile material in an actual forensic case. In another study, Gascho and colleagues (2019) successfully used extended CT scale to differentiate bullets with different jacket materials.

Dual-energy index (DEI) is a simple measure that provides a quantitative metric for a material’s attenuation differences in the two energy ranges measured using DECT. As the energy dependence in attenuation properties is energy-specific (i.e., varies between materials), DEI could be used to discriminate between materials (van der Merwe and Loggenberg 2023).

To conduct this study, several shotgun pellets of various materials were shot into blocks made of ballistic gelatine. We hypothesized that DECT may be capable of at least discriminating steel shot from other pellet materials.

## Materials and methods

### Test firing

Five shotgun pellet materials were selected for this study: lead (Pb), steel (Fe), copper (Cu), bismuth (Bi), and tungsten (W). All the pellets were of commercial origin and shot from factory loaded 12-gauge shotgun ammunition. We aimed to select ammunition with a pellet size of 3 mm in diameter. However, there was some variation in size between different materials, as some pellets were only available in sizes such as 2.75–3.1 mm. The pellet diameter of approximately 3 mm was chosen based on its popularity. Categorized as “bird shot”, this type of ammunition is widely available worldwide and thus would be a common pellet size in real-life shotgun-related incidents. One additional test setting included steel pellets in size of 4 mm in diameter as we wanted to validate the potential effects of pellet size on our results.

To perform the shots, we utilized a Benelli (Urbino, Italy) Montefeltro Synthetic 12/76 semi-automatic shotgun with Aimpoint (Malmö, Sweden) Micro S-1 red dot sight. A 28“ barrel length and cylinder choke were used.

The pellets were shot into five gelatine blocks of 70 mm x 70 mm x 150 mm in size. The gelatine was produced according to the guidelines of Jussila (2004). The blocks were arranged longitudinally toward the shooter. The shooting distance was 20 m. Three consecutive shots per each shotshell type was shot to produce enough pellets into each gelatine block.

The experiments were performed in a restricted area by a researcher with a valid firearms license and long-term experience with firearms (JAJ). Ethical approvals were not required as animals, humans or human cadavers were not involved due to the preliminary nature of the study.

## Radiological imaging

The gelatine blocks were CT scanned at the Department of Radiology, Oulu University Hospital, in Oulu, Finland, using a clinical dual-source system (Somatom Definition Flash, Siemens Healthcare, Forchheim, Germany). Scanning was performed twice to evaluate the robustness of the approach in cases of varying block positioning. For the first scan, the gelatine blocks were positioned longitudinally, while for the second scan, they were positioned transversely. In theory, beam hardening may be slightly more pronounced in the transverse orientation due to the longer x-ray path length through the material, which may affect DEI values.

The blocks were scanned using DECT with the following settings: a rotation time of 0.5 s, pitch factor of 0.5, collimation of 32 × 0.6 mm, and tube voltages of 100 kVp and 140 kVp. A 0.4 mm tin filter was applied to the tube operating at the higher kVp. The quality reference exposure (mAs) values were 200 for the 100 kVp and 386 for the 140 kVp acquisitions, resulting in a volumetric CT dose index (CTDIvol) of 24.75 mGy (dose estimate for 32 cm body phantom). The images were reconstructed using the vendor’s filtered back projection algorithm, yielding a voxel size of 0.504 mm × 0.504 mm × 1.000 mm, with the dual-energy D30f kernel applied. The extended HU scale was also enabled.

Following the image acquisition, the data was analysed using an in-house MATLAB (R2021a, The MathWorks Inc., Natick, MA, 2021) script. This script offers a fully automated pipeline to produce DEI images, segment the pellets, and summarize the mean DEI value of each detected pellet. The DEI image was first calculated using formula

$$\:DEI=\:\frac{{I}_{100\:kVp}-{I}_{140\:kVp}}{{I}_{100\:kVp}+{I}_{140\:kVp}+2000},$$

where *I*_*100 kVp*_ and *I*_*140 kVp*_ are the 100 kVp and 140 kVp images, respectively.

**Fig. 1:**
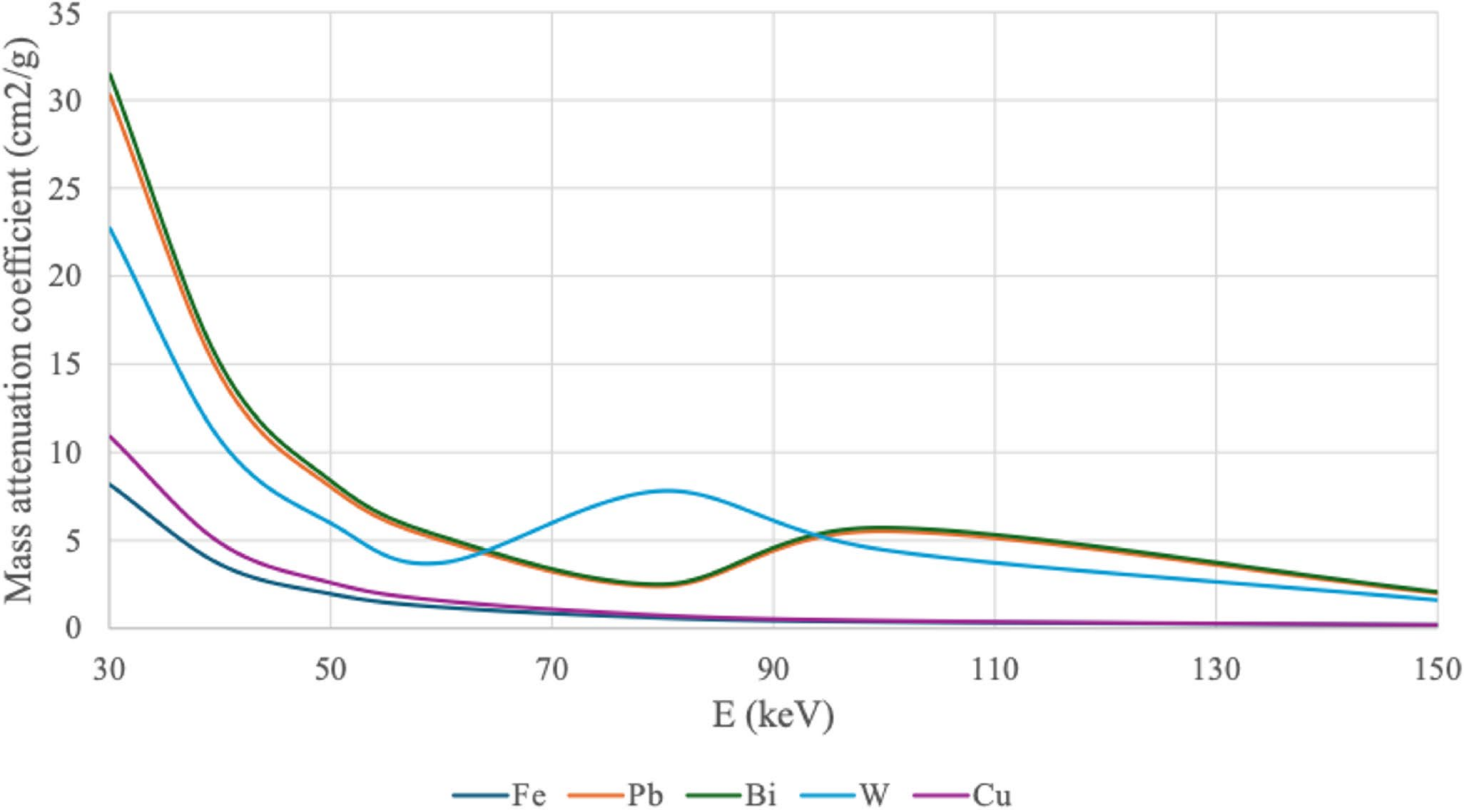
Mass attenuation coefficients of the pellet materials. Mass attenuation coefficients were obtained from the National Institute of Standards and Technology database (Hubbell and Seltzer 1996). Materials are abbreviated according to their chemical symbols: steel (Fe), lead (Pb), bismuth (Bi), tungsten (W), and copper (Cu). The energy range is limited to diagnostically relevant photon energies, from 30 kiloelectron volts (keV) to 150 keV

Fundamentally, the differences in mass attenuation of low-energy and high-energy acquisition dictate the DEI value (the mathematical derivation is provided in the Supplementary information). Consequently, the energy-dependence of the mass attenuation coefficient should indicate which materials could be differentiated with the DEI (Fig. 1).

The pellets were then automatically thresholded from the 140 kV image using a fixed threshold of 2000 HU, i.e., anything above this HU threshold was considered to be a pellet. As these strongly attenuating materials cause severe partial volume effect, a morphological erosion with a disk of 4-pixel diameter was applied to the thresholded segmentation mask to obtain the final segmentation. Each individual pellet was then automatically extracted from the mask by detecting individual connected components (Matlab’s bwconncomp-function). Subsequently, the mean DEI value was documented for each individual pellet.

Box plots were created to illustrate the differences in DEI values between pellet materials, and two-sided Wilcoxon rank sum test was used for numerical comparisons. P values < 0.05 were considered statistically significant.

Finally, to address the robustness of the method with respect to variable pellet size, 20 steel pellets were molded in two gelatine blocks. The first block contained pellets with a 3 mm diameter, and the second pellets with a 4 mm diameter. As the beam hardening effect could be a contributor to potential thickness dependence in DEI values, we selected a lower tube voltage setting for the low kV acquisition to maximize beam hardening, while keeping other measurement parameters the same as for the routine gelatine block measurements. Consequently, for the assessment of thickness dependence, the tube voltage settings were 80 kV and 140 kVp with a 0.4 mm tin filter.

## Results

CT revealed that the test firing had successfully embedded several pellets inside each gelatine block. The total number of pellets for each pellet material was as follows (Fe: 5; Pb: 4; Cu: 13; W: 6; Bi: 8). Pellets were distributed in the blocks approximately 20–120 mm from the entrance. It was evident that the densest pellet materials such as lead, and tungsten showed greatest penetration in gelatine.

**Fig. 2:**
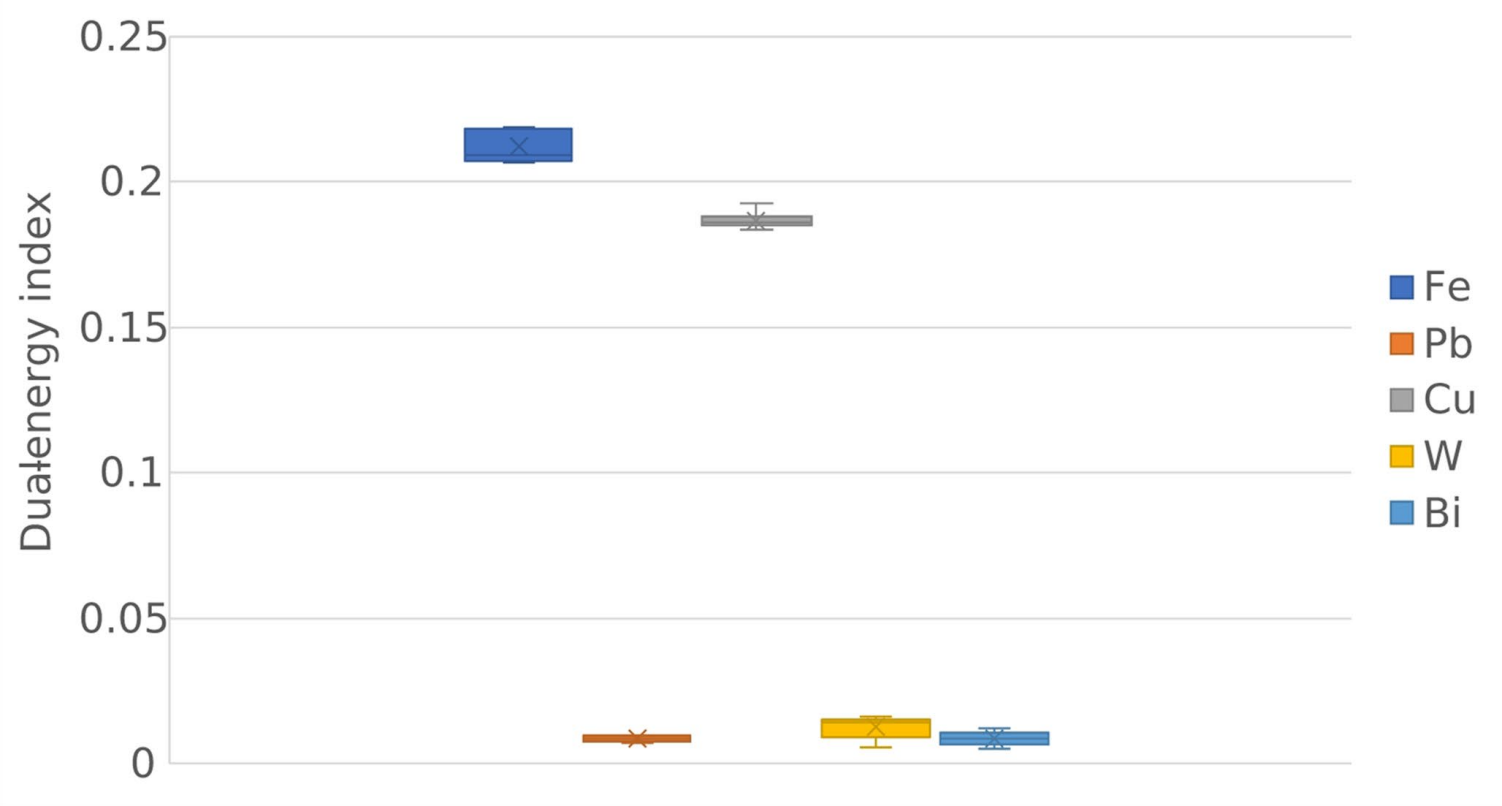
Box plots illustrating the differences in dual-energy index values between pellet materials, i.e., steel (Fe), lead (Pb), copper (Cu), tungsten (W), and bismuth (Bi). Each box represents the interquartile range, with the central line indicating the median and the cross-marker denoting the mean. The DEI clearly differentiates between low and high atomic number materials, with Fe and Cu exhibiting higher DEI values compared to Pb, W, and Bi

The DEI values (mean ± standard deviation) were 0.212 ± 0.006, 0.008 **±** 0.001, 0.187 **±** 0.002, 0.012 **±** 0.004, 0.008 **±** 0.002 for steel, lead, copper, tungsten, and bismuth, respectively. The box plots and two-sided Wilcoxon rank sum test indicated that steel and copper could be reliably differentiated from each other and from the rest of the pellet materials (Fig. 2; Table 1).

**Table 1:** Wilcoxon rank sum test results (p-values). A p-value <0.05 indicates that the sample medians are significantly different

	Fe	Pb	Cu	W	Bi
Fe	**1.000**	<0.001	<0.001	<0.001	<0.001
Pb	<0.001	**1.000**	<0.001	<0.05	**0.869**
Cu	<0.001	<0.001	**1.000**	<0.001	<0.001
W	<0.001	<0.05	<0.001	**1.000 **	**0.058**
Bi	<0.001	**0.869**	<0.001	**0.058**	**1.000**

**Fig. 3:**
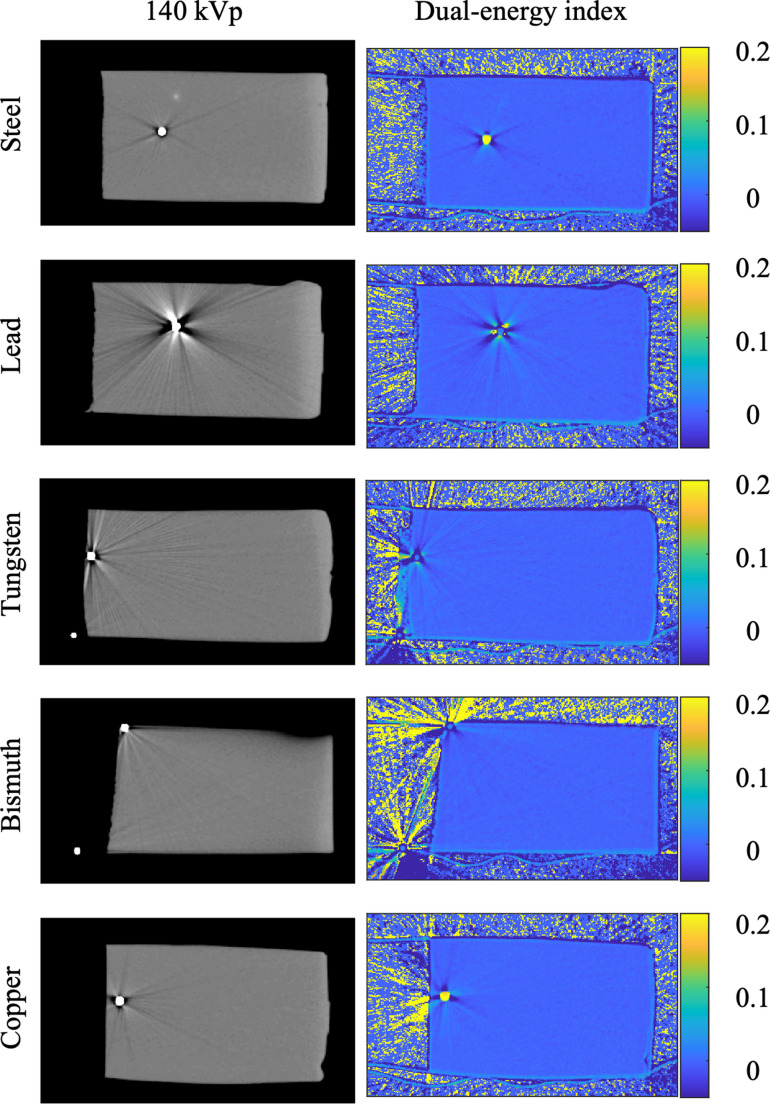
Exemplary reconstructed slices for the 140 kVp acquisition (left) and the corresponding dual-energy index (DEI) value images (right). The DEI images highlight spectral differences between materials, with scale bars indicating DEI values from 0 to 0.2. Beam hardening artifacts are visible in both image types, particularly around high atomic number materials such as lead

The DEI value differences between pellet materials are distinct even visually (Fig. 3). Fe and Cu demonstrate substantially higher DEI values, whereas the Pb, W, and Bi are practically invisible in the DEI image, despite their strong attenuation seen in the 140 kVp image.

**Fig. 4:**
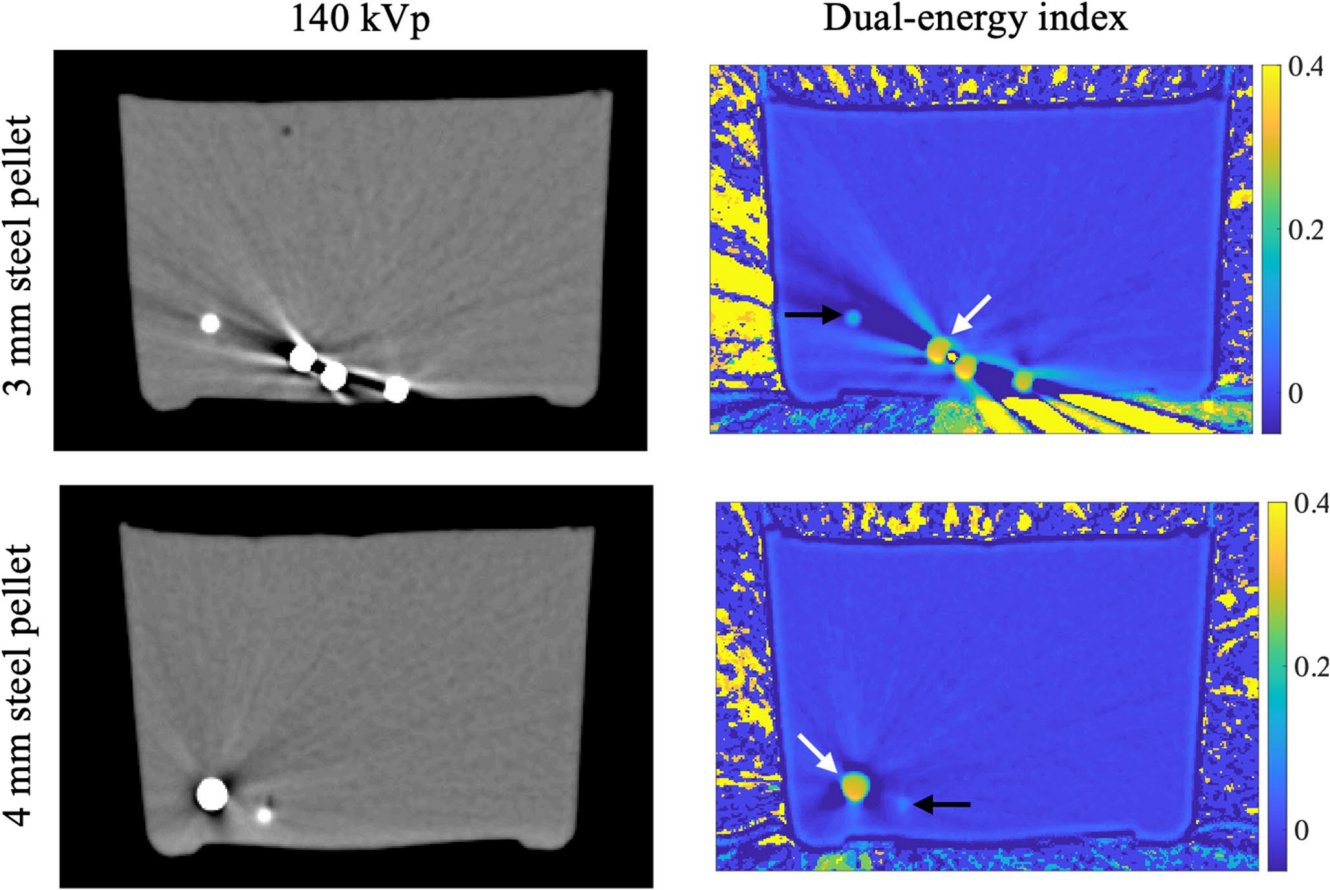
Illustration of the impact of pellet diameter on the dual-energy index (DEI) image. At the center of the pellet (white arrows), the DEI values remain consistent between the two pellet diameters. However, the partial volume effect can be seen to reduce the DEI values of the boundaries of pellets (black arrows). For these black arrows, the centroid of the pellet is in another slice, and thus, the boundary of the pellet is visualized

Regarding the dependency on pellet or shrapnel size, the mean DEI values for steel pellets with 3 mm and 4 mm diameters were 0.300 ± 0.002 and 0.297 ± 0.002, respectively. Consequently, the DEI values differed only by 0.0024 DEI units (0.8%). In the DEI images, the DEI values remained consistent between the two pellet diameters at the center of the pellet (Fig. 4). However, the partial volume effect can be seen to reduce the DEI values of the boundaries of pellets (Fig. 4).

## Discussion

Our research clearly indicated that DECT has the potential to differentiate between specific shotgun pellet materials. This preliminary study suggests that DECT could be a valuable tool in the radiological examination of patients with shotgun-related gunshot injuries. Our findings align with previous research on differentiating projectile materials using DEI values and the extended CT scale (Gascho et al. 2020; Winklhofer et al. 2014). We thus believe that this method could be beneficial for not just clinical forensic cases but also when reconstructing the course of events for postmortem cases when several shotgun pellet types have been used.

Steel and copper shots exhibited distinct DEI values. In contrast, lead, tungsten, and bismuth had similar DEI values to each other and the separation between these materials was not feasible. It can thus be concluded that although DECT can categorize pellet materials, it clearly exhibits some limitations with exact material detection. The high Wilcoxon test p-value of 0.869 for Bi and Pb and the comparable box plots illustrated that these two pellet materials exhibited particularly similar DEI values. This observation aligns well with the nearly identical mass attenuation coefficients of the materials (Fig. 1). Furthermore, differentiating tungsten from bismuth and lead was also observed to be difficult, consistent with the similarities in their mass attenuation coefficients (Fig. 1). Moreover, the strong overall attenuation of these high-atomic-number materials (Z_Bi_ = 83, Z_Pb_ = 82, Z_W_ = 74) compared to those materials with lower atomic numbers (Z_Fe_ = 26, Z_Cu_ = 29) may also contribute to the similarities in DEI values. To improve tungsten differentiation, the DECT protocol could be optimized so that the low-kV acquisition would produce an effective energy of approximately 80 keV, where tungsten exhibits deviating mass attenuation properties compared to Pb and Bi (Fig. 1). However, given the strong attenuation of these materials, care must be taken to prevent signal saturation.

Based on our results, DECT can be potentially utilized for ferromagnetic projectile material detection as already demonstrated by Winklhofer et al. (2014). In this study, steel pellets had distinct DEI values compared to other pellet materials; only copper pellets had relatively similar values to steel ones. This finding is very useful as steel and lead are the most common pellet materials and copper is among the least common ones. Distinguishing between lead, tungsten, and bismuth that are all not ferromagnetic is not a major disadvantage as especially the reliable detection of a ferromagnetic steel shot would be particularly useful in living shotgun injury victims with an urgent need for MRI. More detailed studies are, however, required to create the basis for a reliable methodology to be further utilized in clinical use.

Several studies have recognized the need to increase our understanding of shotgun-related gunshot injuries (Mushtaque et al. 2012). Further studies describing the radiological examination of shotgun wounds in various regions of the body are warranted (Cañas et al. 2007). This would be particularly important for a further understanding of the tendency of pellets to migrate between tissues creating internal damage. In addition, it would be important to be able to distinguish potentially toxic pellet materials from less harmful ones.

We believe that our preliminary study provides an important step for the radiological analysis of shotgun pellets and could be beneficial for both clinical and postmortem cases. We could demonstrate the potential of DECT in foreign material identification and especially categorization and confirm the findings of previous studies that are mainly concentrating on bullet and jacket materials (Gascho et al. 2020; Winklhofer et al. 2014). Our promising results encourage the future use of DECT when victims of shotgun-related incidents are examined in clinical and forensic contexts.

One of the main strengths of the presented approach is its high level of automation for material detection. Our approach was automated and computationally efficient, and could thus be performed on standard computing hardware, such as a laptop. The simplicity and speed of this workflow would make it an accessible tool for broader use in clinical and research settings. However, further studies are required to confirm and develop the methodology presented in this preliminary study. It should also be noticed that the radiation dose of DECT should be optimized for the material identification task in a diagnostic context, in which the as low as reasonably achievable (ALARA) principle should be followed.

The utilized protocol and radiation dose, in particular, warrants a separate discussion. The selected radiation dose aligns with the 2018 American College of Radiology CT Accreditation Program adult diagnostic reference level of 25 mGy (ACR-AAPM-SPR 2024), which was the most recent document at the time of measurements. For improved traceability to this standard and other similar material quantification studies, such as (Jacobsen et al. 2019), we selected this radiation dose level. However, the most recent diagnostic reference levels in Finland are substantially lower than this value. As an example, the dose reference level for abdominal CT in Finland is 12 mGy (CTDIvol).

Our main worry for the present study was the possibility that high noise may hide some of the smaller shrapnels due to the association between image noise and low contrast detectability. However, based on the results, the attenuation from the pellets is so strong that substantially lower doses could be utilized for reliable detection. Furthermore, for diagnostically relevant materials, e.g., iodine, there is strong evidence that the radiation dose level does not have a significant effect on the material quantification accuracy of dual-energy CT excluding diagnostically very low doses (Sartoretti et al. 2022; Jiang et al. 2021). Thus we expect that this dose-independence holds also for the pellet materials. This research question could be examined as a follow-up study.

As a potential limitation, the pellet materials were not confirmed by chemical analysis, but the information was obtained from the ammunition manufacturers. It is possible and even probable that some of the materials were not purely made of just one substance but alloys of two or more substances. This would be the case especially with tungsten pellets as they would be prone to shatter in the pure form. In addition, the pellets of steel shot are not actually steel but made of soft iron. This doesn’t, however, play a significant role from the radiological point of view.

One potential limitation in the pellet analysis could be the sensitivity to the partial volume and beam hardening effects, particularly in relation to the size of the projectile being analysed. Preliminary tests were conducted to explore the impact of pellet size on DEI results. For instance, only minor differences in DEI values were observed between 3 mm and 4 mm steel pellets. As the size of the projectile decreases further, partial volume effect may lead to inaccurate DEI values. This is especially relevant when dealing with very small fragments, where the blending of tissue and material boundaries can skew the results (Guo et al. 2011). However, in shotgun wounds, this should not produce major problems as smallest common pellets are 2 mm in diameter, and in general, pellets tend to remain intact.

On the other hand, the presence of larger metallic objects, such as more sizable pellets or shrapnel, may introduce beam hardening artifacts. However, shotgun pellets rarely are more that 4.5 mm in diameter and in extreme cases their diameter may be up to 8 mm. Beam hardening, known to cause inaccuracies in HU measurements, could similarly affect DEI values. Consequently, beam hardening may introduce another variable that could complicate the interpretation of DEI data (Kanatani et al. 2021). Future research should systematically investigate the influence of both small and large fragment sizes on DEI accuracy to better understand the impact of partial volume and beam hardening before considering the diagnostic adoption of the presented method.

Another important consideration relates to the thresholding method used for pellet segmentation. A limitation of our automated segmentation approach is the use of a fixed 2000 HU threshold, which may not be sufficient to distinguish shotgun pellets from other dense foreign objects in clinical or forensic imaging, such as dental prostheses or metallic implants. While this threshold was effective in our controlled gelatine block environment, real-life cases may require additional criteria. Our segmentation code is flexible in this regard. It identifies each connected component individually, which would allow users to inspect and verify each segmented object separately. The workflow also allows integration of additional shape or size-based filtering, which can help restrict the analysis to fragments with dimensions and morphology consistent with shotgun pellets, reducing the inclusion of irrelevant objects in diagnostic and forensic imaging.

The medico-legal significance of this study can be highlighted from several perspectives. First, in medico-legal systems where autopsies cannot necessarily be performed on an immediate basis, postmortem CT may constitute a means of providing the police with relevant pieces of information rapidly, for example, on the material of shotgun pellets associated with a homicide case. Second, in mass disasters, the resources for performing full medico-legal autopsies in a timely manner may be limited. While providing an efficient method to document findings, CT scans may also be used for the rapid extraction of data regarding, for example, projectile materials deposited in a victim. Third, in routine medico-legal autopsies, manually searching for pellets in tissues may be time-consuming and not all pellets can be retrieved despite all efforts. This may increase the possibility of error, in particular in cases where several pellet types have been used together, and not all can be analyzed. Fourth, among living patients with shotgun injuries, imaging often is the only means of evaluating the properties of the pellets embedded in the body.

## Conclusion

This study demonstrated the potential of DECT in the differentiation between shotgun pellet materials. Steel and copper pellets could be reliably differentiated from other materials, whereas lead, tungsten, and bismuth exhibited similar dual-energy index values and could not be distinguished from each other. As such, the findings suggest that a DECT-based analysis of pellet materials could be a valuable tool in the medico-legal investigation of shotgun-related fatalities and in trauma centres treating patients with shotgun wounds. In addition, this method could be helpful in postmortem cases with potential trauma from several pellet types.

## References

[CR1] American College of Radiology, American Association of Physicists in Medicine, Society for Pediatric Radiology (2024) ACR-AAPM-SPR practice parameter for diagnostic reference levels and achievable doses in medical x-ray imaging

[CR2] Byrne R (2023) Review of the legal and regulatory history of lead in hunting. Wildl Soc Bull 47:e1465

[CR3] Cañas A, Almodóvar L, Lima P, Buendía J (2007) Perdigón cardiaco en el septo interventricular [Cardiac shotgun pellet in the interventricular septum]. Rev Esp Cardiol 60(9):994–995 17915160 10.1157/13109657

[CR4] Ditkofsky N, Colak E, Kirpalani A et al (2020) MR imaging in the presence of ballistic debris of unknown composition: a review of the literature and practical approach. Emerg Radiol 27:527–532 32418149 10.1007/s10140-020-01781-6

[CR5] Gani F, Sakran J, Canner J (2017) Emergency department visits for Firearm-Related injuries in the united states, 2006–14. Health Aff 36:1729–1738 10.1377/hlthaff.2017.0625 28971917

[CR6] Gascho D, Zoelch N, Richter H, Buehlmann A, Wyss P, Schaerli S (2019) Identification of bullets based on their metallic components and X-Ray Attenuation characteristics at different energy levels on CT. AJR Am J Roentgenol 213:W105–W113 31120788 10.2214/AJR.19.21229

[CR7] Gascho D, Zoelch N, Deininger-Czermak E et al (2020) Visualization and material-based differentiation of lodged projectiles by extended CT scale and the dual-energy index. J Forensic Legal Med 70:101919 10.1016/j.jflm.2020.101919 32090974

[CR8] Golden NH, Warner SE, Coffey MJ (2016) A review and assessment of spent lead ammunition and its exposure and effects to scavenging birds in the United States. Rev Environ Contam Toxicol 237:123–191 26613991 10.1007/978-3-319-23573-8_6

[CR9] Guo X, Wang B, Duan S et al (2011) The influence of partial volume effect on the finite element modeling of bone, *2011 IEEE International Symposium on IT in Medicine and Education*, Cuangzhou, 477–480

[CR10] Hubbell J, Seltzer S (1996) X-Ray Mass Attenuation Coefficients. Accessed: Apr. 19, 2018. [Online]. Available: https://www.nist.gov/pml/x-ray-mass-attenuation-coefficients

[CR11] Jacobsen MC, Cressman ENK, Tamm EP, Baluya DL, Duan X, Cody DD, Schellingerhout D, Layman RR (2019) Dual-Energy CT: lower limits of iodine detection and quantification. Radiology 292:414–419 31237496 10.1148/radiol.2019182870 PMC6694721

[CR12] Jiang X, Yang X, Hintenlang DE, White RD (2021) Effects of patient size and radiation dose on iodine quantification in dual-source dual-energy CT. Acad Radiol 28:96–105 32094030 10.1016/j.acra.2019.12.027

[CR13] Juntunen M et al (2020) Framework for photon counting quantitative material decomposition. IEEE Trans Med Imaging 39:35–47 31144630 10.1109/TMI.2019.2914370

[CR14] Jussila J (2004) Preparing ballistic gelatine–review and proposal for a standard method. Forensic Sci Int 141(2–3):91–98 15062946 10.1016/j.forsciint.2003.11.036

[CR15] Kanatani R, Shirasaka T, Kojima T et al (2021) Influence of beam hardening in dual-energy CT imaging: Phantom study for iodine mapping, virtual monoenergetic imaging, and virtual non-contrast imaging. Eur Radiol Exp 5:18 33903993 10.1186/s41747-021-00217-1 PMC8076398

[CR16] Kara M, Polat H, Ay S (2008) Penetrated shotgun pellets: A case report. Eur J Dentistry 2:59–62 PMC2633155 19212510

[CR17] Mushtaque M, Mir M, Bhat M et al (2012) Pellet gunfire injuries among agitated mobs in Kashmir. Ulus Travma Acil Cerrahi Derg 18:255–259 22864719 10.5505/tjtes.2012.47639

[CR18] Nee N, Inaba K, Schellenberg M et al (2021) Retained bullet fragments after nonfatal gunshot wounds: epidemiology and outcomes. J Trauma Acute Care Surg 90(6):973–979 33496545 10.1097/TA.0000000000003089

[CR19] Pinotti E, Santurro L, Famularo S, Uggeri F (2017) Surgical removal of radiolucent lodged bullet fragment 5 years after a shotgun injury. Open J Clin Med Case Rep 3(14):1286

[CR20] Rowe WF, Hanson SR (1985) Range-of-fire estimates from regression analysis applied to the spreads of shotgun pellet patterns: results of a blind study. Forensic Sci Int 28:239–250

[CR21] Sartoretti T, Mergen V, Jungblut L, Alkadhi H, Euler A (2022) Liver iodine quantification with Photon-Counting detector CT: accuracy in an abdominal Phantom and feasibility in patients. Acad Radiol 30:461–469 35644755 10.1016/j.acra.2022.04.021

[CR22] Symons R, Cork TE, Lakshmanan MN et al (2017) Dual-contrast agent photon-counting computed tomography of the heart: initial experience. Int J Cardiovasc Imaging 33:1253–1261 28289990 10.1007/s10554-017-1104-4

[CR23] Utkan A, Koçer B, Gencer B, Özkurt B (2020) Unexplained pellets in heart after shotgun wound through the hip: A case report. J Surg Med 4:248–250

[CR24] van der Merwe F, Loggenberg E (2023) Dual-energy index variation when evaluating the potential ferromagnetism of ex vivo bullets. South Afr J Radiol 27:1 10.4102/sajr.v27i1.2701 PMC10696535 38059118

[CR25] Weiss D, Tomasallo CD, Meiman JG et al (2017) Elevated blood lead levels associated with retained bullet Fragments - United states, 2003–2012. MMWR Morb Mortal Wkly Rep 66(5):130–133 28182606 10.15585/mmwr.mm6605a2 PMC5657964

[CR26] Winklhofer S, Stolzmann P, Meier A, Schweitzer W et al (2014) Added value of dual-energy computed tomography versus single-energy computed tomography in assessing ferromagnetic properties of ballistic projectiles: implications for magnetic resonance imaging of gunshot victims. Invest Radiol 49:431–437 24566289 10.1097/RLI.0000000000000032

